# A Ubiquitous NFC Solution for the Development of Tailored Marketing Strategies Based on Discount Vouchers and Loyalty Cards

**DOI:** 10.3390/s130506334

**Published:** 2013-05-14

**Authors:** Francisco Borrego-Jaraba, Pilar Castro Garrido, Gonzalo Cerruela García, Irene Luque Ruiz, Miguel Ángel Gómez-Nieto

**Affiliations:** Department of Computing and Numerical Analysis, Albert Einstein Building, Campus de Rabanales, University of Córdoba, Córdoba E-14071, Spain; E-Mails: fborrego@uco.es (F.B.-J.); pcgarrido@uco.es (P.C.G.); gcerruela@uco.es (G.C.G.); mangel@uco.es (M.A.G.-N.)

**Keywords:** near field communication, mobile phones, vouchers, m-coupons, marketing, loyalty cards

## Abstract

Because of the global economic turmoil, nowadays a lot of companies are adopting a “deal of the day” business model, some of them with great success. Generally, they try to attract and retain customers through discount coupons and gift cards, using, generally, traditional distribution media. This paper describes a framework, which integrates intelligent environments by using NFC, oriented to the full management of this kind of businesses. The system is responsible for diffusion, distribution, sourcing, validation, redemption and managing of vouchers, loyalty cards and all kind of mobile coupons using NFC, as well as QR codes. WingBonus can be fully adapted to the requirements of marketing campaigns, voucher providers, shop or retailer infrastructures and mobile devices and purchasing habits. Security of the voucher is granted by the system by synchronizing procedures using secure encriptation algorithms. The WingBonus website and mobile applications can be adapted to any requirement of the system actors.

## 1. Introduction

The ambient intelligence paradigm (AmI) aims to develop environments able to interact with the user in an autonomous way in order to make life easier for people in different fields [1]. A technology that facilitates the achievement of the AmI objectives is Near Field Communication (NFC) [2]. NFC simplifies the users' interactions with the context-aware services offered in intelligent environments, also promoting a new interaction model, called “*touch paradigm*”. This paradigm allows the construction of a complete information environment, where ubiquitous applications allow users to obtain any information or services from the surrounding objects, which are augmented with RFID Tags, just by touching them with a NFC device.

Another important aspect that favors the development of intelligent environments is the current and future evolution of mobile devices and the number of terminals around the world [3]. The increase in the number of phones in the world has followed an exponential curve, from about one thousand million mobile phones worldwide in 2001, to about five thousand million in 2011, which has led to the development of new applications based on NFC, applied to a wide variety of services [4].

In addition to the obvious payment and security applications [5–8], NFC has provided a wealth of applications in transport [9–12], advertising and promotion of tourism businesses [13–17], ambient assisted living (AAL) [18–21], elderly care [22–25], shopping [26,27], authentication [28–31], university of things [32,33], and oriented to a wide variety of scientific, as well as business areas [34,35].

Besides this, the current global turmoil is forcing citizens to save money and to seek cheaper prices when they purchase products and services. Thus, marketing and loyalty techniques are changing, helped by the overwhelming power of smart phones over traditional mobile phones, creating the concept of m-coupons or mobile coupons.

In the market, we can find some applications that already work with m-coupons. Chen *et al.* [36] propose an m-coupon sharing scheme that uses mobile devices to distribute coupons among existing social networks, increasing m-coupons exchange. In this scheme, the company first selects targeted members and then it sends a virtual coupon book and a virtual sharable coupon book to each of these targeted members. The targeted member is encouraged to forward the sharable coupons to his/her peers.

Dominikus and Aigner [37] propose a new form of coupons called mCoupons which can be downloaded from a poster or a newspaper equipped with a passive NFC device to a mobile phone. With this mobile device the user can then cash in the mCoupon at the cashier. They also have described two protocols for secure mCoupons with different security features.

Will *et al.* [38] describe a framework to exploit social network connections for targeted campaign delivery in order to support local retailers in enhancing customer communication. They have implemented a client application enabling end users to find relevant coupons and offers and retrieve them after completing specific tasks.

Other applications such as Groupon [39] offer deals that last about a day in most of the markets around the World. This type of offer is known as “deal of the day”. The user pays first and then he/she gets the coupons to be exchanged at the indicated shop. These types of deals are exchanged through traditional paper coupons or through their mobile application using QR codes [40], that the partner shop reads using a QR reader or checking and writing down the coupons code. Other applications like LetsBonus [41] or Groupalia [42] also use QR codes for redemption of the coupons. However, Coupies application [43] uses NFC technology to redeem the coupons. The buyer only has to bring his/her NFC device to Coupies Touch Points. These Coupies Touch Points are coded stickers which are located at the points of sale. In addition, this sticker has a QR code for those users who do not have mobile devices equipped with NFC technology.

Although some existing systems are oriented to the elimination of the traditional paper voucher using new technologies and mobile devices, some challenges are as yet unsolved. Valuable aspects required to pervasive systems, such as mobile applications, are not considered, as in the following:

– Security: vouchers are managed in mobile devices as tangible coupons. Once the voucher is bought from the provider website, security can only be checked by the PoS (point of sale) system. Aspects regarding the loss of vouchers, error in the redemption process, error in the manipulation process by the user or device, fraudulent copying, *etc.*, are not well solved yet.
– Voucher type: most of the current systems usually consider a restricted type of vouchers: discount coupons; that users must pay in advance and vouchers can be later redeemed at a specific establishment. However, the providers and shops/retailers would need a wider type of voucher, depending on the characteristics of the product or service, voucher price, the desired targets or user characteristics, *etc.* For instance: coupons not necessarily being paid in advance, vouchers that could include a set of redemptions (bonds), vouchers that could be a kind of prize that the user accumulates until the user has a large enough number of them to be redeemed by a product or service, *etc.*
– Architecture: websites are in charge of the voucher publicity, provision and payment, and the commerce infrastructure, if it exists, is in charge of the checking and exchange. Thus, current proposals are not open to the requirements of the voucher providers, where providers systems' can be involved in the process. The voucher provider is the company that provides the marketing campaign, which can be the same shop in which the coupons will be redeemed or a manufacturer that trades the product considered in the voucher.
– Loyalty cards: consideration of the loyalty cards corresponding to providers and/or commerces have not been taken into account. Although some existing applications manage loyalty cards, these applications do not integrate in a unique solution all type of vouchers and loyalty products.
– Adaptive vouchers: current systems show shortcomings in the management of user preferences in order to offer and supply vouchers. Because of the above mentioned paradigm “deal-of-the-day”, they are offering just a few vouchers each day that do not adjust to the user preferences but only to the city selected for the redeem process.
– Getting vouchers: current systems supply vouchers mainly through the website. This does not adjust to a pervasive system. Thus, the process of getting vouchers should be distributed by getting them anywhere easily with a smart phone.

This paper presents a framework aimed to solve all the aforementioned problems. The framework, called WingBonus [44], is not only an application oriented to mobile coupons management. WingBonus is an ecosystem that can be fully tailored to providers, retailers and users' requirements. Thus vouchers publicity, sourcing and redemption can be performed by any infrastructure owned by any of the actors participating in the different processes. Besides, security of vouchers is always granted through unique secure keys, information encryption and double or triple secure validation using WingBonus, providers' and/or retailers' infrastructure.

WingBonus supports any type of vouchers, including the ones oriented to attract user loyalty through discounts on each purchase of a product or service. This kind of voucher is called a chit and its management is very similar to loyalty cards management that is also considered by WingBonus, thus integrating in a unique system all type of vouchers.

Moreover, the WingBonus Mobile Application is fully adapted to providers and users' requirements. Providers' campaigns are automatically displayed in the mobile and web applications. The system can even be personalized to providers in the same way as the systems that are owned by providers.

Finally, WingBonus integrates QR and NFC technologies, allowing the sourcing and redemption of any kind of vouchers using the available technology: smart posters, NFC readers and QR readers for retailers, environment and user devices.

The paper is organized as follows: Section 2 describes the system and environment infrastructures. Section 3 presents the architecture of the system, describing the main components and the processes between the actors, showing how the solution can be tailored for different levels of security and different characteristics of retailer infrastructure. The WingBonus website is described in Section 4 and the WingBonus mobile application in Section 5. Finally, in Section 6 we discuss the results obtained and we show the advantages of WingBonus compared to classical marketing campaigns based on paper vouchers and other existing systems where NFC technology is not considered.

## 2. Description of the System

In this paper, we propose an ecosystem called *WingBonus*. WingBonus is in charge of disseminating, distributing, sourcing, validating and managing loyalty cards and electronic vouchers using mobile technologies.

### 2.1. System Technology

WingBonus is composed of two main systems: the WingBonus website and the WingBonus mobile application. WingBonus website has been developed using as HTML5 [45] and CSS3 [46] ensuring its adaptation to the standards recommended by W3C [47]. We have followed one-page development paradigm using Javascript and Backbone.js [48] complemented by JQuery [49] and libraries such as Underscore [50] of Require.js [51]. The server side processing has been developed using PHP.

The WingBonus mobile application communicates with the server side through web services. In this proposal, we have implemented RESTful web services [52]. For service representation we have used JSON format [53] in order to implement communications between the server and mobile applications in the synchronization process. The database engine used has been MySQL on the server and SQLite in the mobile application.

In order to provide security to the information stored on both the server and the mobile device, we have decided to encrypt the information using a symmetric cipher called Blowfish [54]. To date, no effective Blowfish analyzer has been found, maybe because more importance has been given to the decoding of larger blocks using programs such as AES [55] or Twofish [56]. Blowfish uses 64-bit blocks and a key size from 32 bits to 448 bits. Another important aspect is that Blowfish is not patented. The algorithm is publicly available and can be freely used by anyone.

The WingBonus mobile application currently runs on the Android [57] and RIM [58] operating systems. Regarding environment infrastructure WingBonus considers different types of RFID Tags [59,60], several types of NFC readers, such as the ACR 122U [61], and any QR reader, to redeem vouchers if the user or the establishment does not use NFC.

Thus, a driver and an API have been developed in Java. This API implements the communication between a mobile device and a reader at the point of sale (PoS). This communication allows: (a) redemption of vouchers, (b) distribution of vouchers, and (c) transactions with loyalty cards. Furthermore, this API enables communication processes using NFC and QR codes. This communication process is carried out as follows:

– NFC communication: the user touches the NFC reader located at the PoS with his/her mobile device equipped with NFC to redeem a voucher. At this time, the communication process is carried out. The PoS reads the information from the mobile phone and sends it to the server to verify the validity of the voucher. Then, the PoS sends the user the answer through the reader. This process is similar for distributing vouchers at the PoS and transactions with a loyalty card.
– Non-NFC communication: the user shows the QR-code of the voucher at the PoS and the communication process is carried out. PoS reads the information through QR-reader and it sends the information to the server to verify and validate the voucher. This process is similar to transactions with a loyalty card.

### 2.2. Environment Infrastructure

Our pervasive system is open to a wide range of infrastructures in order to manage the full voucher processing chain. Different actors are considered by the systems: (a) providers, any manufacturer promoting a product or service, (b) retailers, any shop or commerce that either promotes a product and/or participates as PoS (point of sale) in any of the voucher processes, and (c) users, any user selecting and redemption of any kind of vouchers and managing loyalty cards.

All vouchers and loyalty card processes are related to these actors and their characteristics depend on the existing environment infrastructure. WingBonus permits voucher advertising and sourcing to be performed from provider and retailer infrastructure, if existing. Thus, provider and retailer websites can be used for promoting and sourcing vouchers to users as if the process is performed from the WingBonus website. In addition, vouchers can be sourced to users from NFC readers or any Smart Poster including RFID Tags or QR codes distributed by providers or retailers. The mobile application is in charge of the selection and sourcing of the voucher and sending this information to the server to be validated.

In addition, the WingBonus website manages all kind of vouchers as well as providers' and retailers campaigns. Users connect to the web portal, selecting and downloading the desired vouchers or loyalty cards. Moreover, WingBonus website validates the voucher sourcing carried out by providers, retailers and smart posters.

Moreover, in the voucher redemption and validation processes and card operations different actors are also involved depending on the actors involved in the sourcing process. Vouchers are always redeemed in the PoS using the retailer infrastructure, this process being controlled and validated by the WingBonus server. In addition, provider and retailer website may participate if they are in charge of the voucher sourcing. Depending on PoS infrastructure the validation and redemption of vouchers can be carried out using different security levels. As we describe below in the paper, the security level in the voucher management is related to characteristics of the advertising campaign, type of voucher, use or not of NFC reader in the PoS, *etc.*

## 3. Architecture of the System

### 3.1. Types of Vouchers

**Figure 1.**
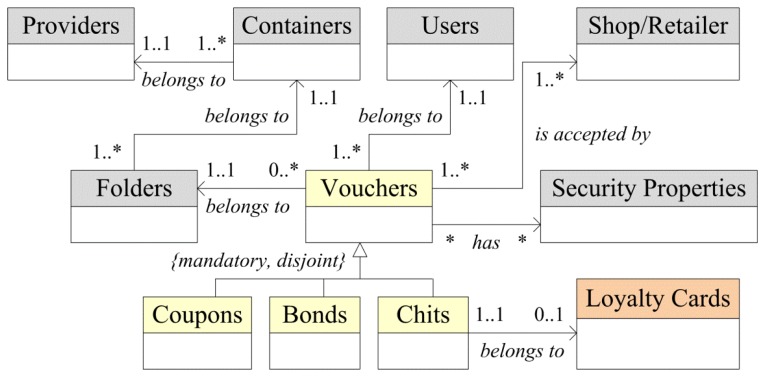
Class diagram corresponding to voucher information.

Figure 1 shows some of the main information classes considered for the representation of vouchers' information. Three different types of vouchers are managed by the systems, as follows:

– Coupons: a coupon allows the end customer to acquire a product or a service with a certain discount. Current solutions require printed coupons, or, in the best-case scenario, the need to install several apps in the mobile device. WingBonus gathers and stores all kind of coupons that the user can use when and where he/she wants, in a simple and safe way.
– Bonds: a bond is a set of coupons. The user can take advantage of a bulk purchase of coupons obtaining a notable discount because WingBonus offers capabilities for bonds management.
– Chits: If retailers and providers want to attract customers to purchase the same product or service more than once, customer loyalty and savings are encouraged. WingBonus creates the concept of “chit”. A “chit” is a small reward for the purchase of a product or service. WingBonus manages user chits and it allows users to exchange them at will for new products or services or even discounts.

Offers and providers' promotional campaigns are managed through *Containers*. Containers allow providers to group some vouchers, satisfying their business policies. Moreover, containers can be classified in *Folders*, the vouchers being assigned to a specific folder. A folder is used to define specifics providers' campaigns. Thus, a provider can distribute their vouchers in different business activities and different campaigns using different provider's images, requirements, and promotional, business and marketing policies using the same user environment.

In addition, WingBonus functionality includes the management of electronic loyalty cards. The user can manage any number of loyalty cards in his/her mobile phone. Furthermore, a loyalty card is managed like a folder, restricted to storing only chits. When a user buys any product or service corresponding to a specific provider or retailer he/she receives economic or symbolic rewards that are accumulated in the loyalty card corresponding to the retailer or provider. Afterwards, the user can exchange those rewards wherever he/she wants for other products or services, always following the requirements of the loyalty card.

Properties defined in containers and folders allow for use of different icons, images, *etc.*, and to define the different type and characteristics of the voucher for each of them. WingBonus manages this information to generate tailored interface in the mobile application as we will describe later.

Users can select vouchers, these vouchers being assigned unequivocally to each user to be later sourced to the mobile application. These vouchers are exchanged in retailers or shops, any point of sale (PoS), defined and accepted previously when a voucher is created. Retailers and providers can be the same actors, for example in the case of a shop offering a loyalty card to its customers.

Security of the vouchers is defined by the providers depending on the characteristics of the marketing and business campaigns of the vouchers. Voucher security is managed depending on the user activity and the voucher characteristics. Thus, voucher security is defined for:

– Voucher selection: when a user chooses a voucher in order to be downloaded afterwards in his/her mobile phone.
– Voucher sourcing: in this process the marked voucher is downloaded to the mobile phone.
– Voucher redemption: the voucher is exchanged in an accepted retailer.
– Voucher transfer: the voucher is transferred from one user to another.

For each of these functionalities the system considers security properties about:

– User identification: the user must be identified in the system. When the process is being carried out, the system will check the user granting or rejecting the request.
– Voucher properties: the properties of each voucher, defined individually for each one of these processes.
– Environment/scenario: vouchers can be allocated, as will be described below, and therefore sourced from different media or infrastructures. Thus, a voucher can be sourced from smart posters, store readers, as well as WingBonus or provider websites. Besides, users' mobile phone or environment can determine the communication infrastructure (feasibility of WiFi and GPRS communication, for instance). These characteristics can be defined in the security properties of the vouchers in order to adjust the security level for each of the processes.

### 3.2. Voucher Lifecycle

**Figure 2.**
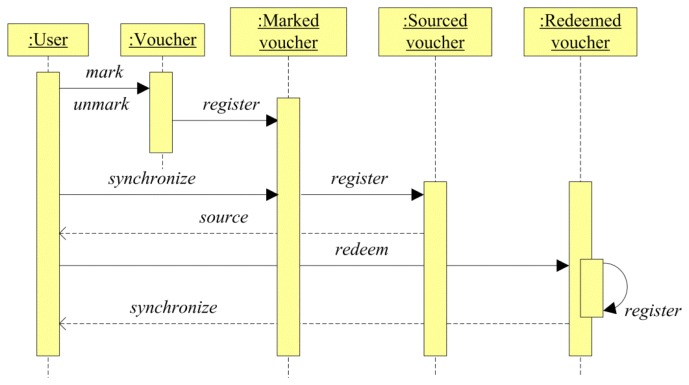
Sequence diagram showing the voucher lifecycle.

Vouchers are defined at different status depending on the process performed on them by the actors participating in the system. Figure 2 shows a sequence diagram of the voucher lifecycle and the process in charge of moving a voucher from one status to another.

First of all, vouchers are defined and set at the disposal of the users for their selection. Characteristics of the vouchers such as due date, total number of sourcings, total selection number by user, number of sourcings by user and time, deadline for the exchange, *etc.*, are set in the voucher definition.

When a user marks a voucher, it goes to the “Marked voucher” status and a unique identification is given for it, considering the user and voucher identification. Thus, users can mark any quantity of voucher at anytime and those vouchers are stored in the system, waiting to be provisioned to the user device.

The sourcing of a voucher can be performed automatically or on user demand. When a voucher is sourced to the user mobile, the voucher passes to the “Sourced voucher” status. The voucher sourcing is performed through a synchronization process in the WingBonus mobile application.

Finally, the voucher passes to the “Redeemed voucher” status when the user redeems the voucher in one of the accepted stores or locations. The synchronization during the redemption process can be carried out in several ways depending on user and shop infrastructure.

WingBonus's database stores information about the whole voucher lifecycle. Vouchers have a unique identification in each status and all voucher statuses are related to each other. The synchronization process validates the mobile and server databases, maintaining one unique and congruent information flow over vouchers in their different status.

Depending on voucher security properties, mobile and server databases can store a different image of user vouchers. For instance, users can source a voucher with low security requirements (*i.e.*, from a smart poster), and users can even redeem those vouchers in shops with the security level established by themselves (for instance, vouchers spread up in smart posters around a city, chits of their own shop, *etc.*). Even in this case, each voucher is uniquely identified in the mobile database, therefore two identical vouchers do not exist. Just when the synchronization is performed, vouchers are stored in the server database and mobile vouchers are updated in order to relate and unify both databases.

### 3.3. Actors and Infrastructure

It can be observed that the system considers three types of actors: providers of vouchers, retailers or shops and users or customers. Providers are companies having marketing or promotional vouchers or loyalty card campaigns. Retailers are the stores or shops where customers earn and redeem vouchers and can play the role of providers in some cases, and users are the customers using the application.

WingBonus is currently open to any provider infrastructure as well as shops' business policies. Therefore, the system allows any infrastructure to be used for any of the process involved in the voucher management.

Vouchers can be selected and sourced from: (a) the WingBonus website, (b) the provider's website, (c) shops' readers, and (d) smart posters.

The user can select and source vouchers from the WingBonus website from a laptop or mobile application. In this case, the system has full control of the information and the process. Besides this, providers can publish vouchers on their own websites and users can select and source those vouchers with or without using the WingBonus system at that moment. The providers define how these vouchers operate through the vouchers' security properties, taking into account the infrastructure of the PoS where the vouchers may be redeemed.

Furthermore, vouchers can be selected and sourced from shop readers, for instance when a loyalty card is used, or from any smart poster displayed anywhere. In this case, vouchers' security is defined by the provider, it being in the low-high range depending on the provider campaign. The security level affects different voucher and infrastructure characteristics that are defined by the provider when an advertising campaign is trade. Thus, providers define how a voucher can be sourced (from WingBonus website, provider website, smart poster and/or NFC reader at the shop), how many vouchers can be sourced (total number of vouchers, total number of vouchers per day, total number of vouchers issued to each user, number of voucher per day for each user, *etc.*), if the voucher should be validated beforehand to be redeemed, how the vouchers should be redeemed (using a NFC reader, using a Smart Posters, both, *etc.*).

User infrastructure is also open. Although WingBonus is a system conceived for using NFC technology, due to the fact that currently some users do not own NFC devices, the system also allows the use of QR codes for the selection of the vouchers. The sourcing also can be performed using any communication media: Wi-Fi, GPRS, Bluetooth, MMS or connecting the phone to the computer.

Depending of the security properties of the vouchers, the redemption process also can be performed using different infrastructures. Thus, retailers can use Tags, NFC or QR readers as media control, and voucher validation can be performed by the retailers, providers and/or WingBonus in real time. In any case, as described below in this paper, full control of vouchers is managed by WingBonus during the whole life of the voucher.

### 3.4. Voucher Management

Security, infrastructure, actors and voucher's lifecycle are fully related in WingBonus in order to offer an open system. Indeed, WingBonus is not properly a system but rather a framework allowing any provider or retailer to develop their own publicity, marketing, loyalty and marketing campaigns using a fully tailored mobile application and considering any voucher characteristics and security requirements.

**Figure 3.**
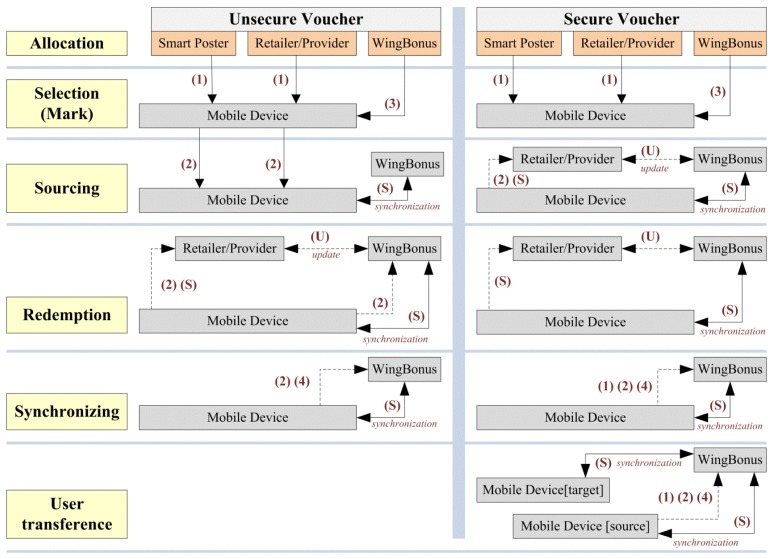
Flow of information between the system actors for secure and unsecure vouchers.

Management of vouchers (see Figure 3) depends on the security properties defined for each voucher type involved in an advertising campaign. Security level is defined for each of the main processes performed with the vouchers by the system and users, that is: selection, sourcing, redemption and transference among users.

The security level defined by a process determines the security of the later process in the voucher lifecycle. Security level takes into account user identification as well as the grants to perform a process by the user. Furthermore, security level determines in what step of the voucher lifecycle the system grants the voucher authentication and therefore the system stores all the information about the voucher, process and participating actors.

Figure 3 shows the different kind of flows depending on the voucher security and the actors participating in the different phases of the voucher lifecycle. Thus, flows (1) and (2) represent the information gathered by the mobile application from smart posters and retailers (or providers), respectively; (3) represents the information managed by WingBonus server that it can be marked and sourced to users, and (4) the information sent to the WingBonus server by the mobile application in order to be validated and later to process a synchronization between server and mobile applications; finally the flow of synchronization (S) is in charge of matching information between both sides, mobile and server, maintaining the consistency of the information, and the flow label as (U) represents the communication between retailers (and providers) with the server application in order to share information about any information transference between users and retailers (and providers) and users with WingBonus server.

Vouchers can be defined as unsecure for sourcing (see Figure 3). Thus, identified and unidentified users can get vouchers from any source: provider, store, smart poster (flows 1, 2). Selected and sourced vouchers from WingBonus website (flow 3) are stored in the system database as Figure 3 shows. When users access the retailer/provider website, they can select and download vouchers, in this process, provider's website sends a request to a WingBonus server service (flow U), WingBonus's system being in charge of sourcing the voucher to the user and store the information in the system database. Furthermore, the user can get vouchers from smart posters.

Smart posters can store full or partial information of the vouchers depending on the source security property (see Figure 3). If the voucher is secure for sourcing, the partial information got from the smart poster is sent to the system and the full voucher's information is sent back to the user (flow S). These vouchers cannot be sourced in the mobile device until synchronization with the system is performed; however, temporary information about the selected voucher is stored on the phone.

If the voucher is unsecure for sourcing, the mobile application generates a unique voucher identification, which is temporary until the next synchronization is performed. Stores are considered as smart posters although reading infrastructure was used.

Before the later process vouchers always have to be validated by means of a synchronization process. Hence, vouchers are not previously synchronized (flows 1 and 2) and vouchers not previously validated are sent to WingBonus server being validated (or not) and a synchronization process is performed.

Redemption process also can be performed by identified and unidentified users. In this process the user shows the voucher in the store to be exchanged for a product or service. If a high security is not defined for redemption, the user can exchange a source voucher in the allowed shop (see Figure 3). For that, information of allowed stores where the voucher can be redeemed must be stored in the mobile phone, otherwise synchronization is needed, and at this moment the voucher is validated, or not, as well as updated in the mobile phone and stored in the server by means of a new synchronization.

If any security level is defined for redemption, the voucher must be validated prior to being redeemed. In this process, validation codes are sent to the mobile application in order to generate the QR code or NFC keys corresponding to the voucher. The validation process can be performed by different means: (a) the user touches the store's NFC reader connected to the store's computer that communicates with WingBonus's website using a specific service, (b) the user reads a specific Tag (QR or NFC) with his/her mobile phone and use Wi-Fi or GPRS to communicate with the WingBonus system. Finally, the redemption of the voucher is confirmed and communicated to all the involved actors. Retailers will receive information about the vouchers managed by WingBonus at any time depending on the established business agreements.

The most secure way to redeem vouchers is through NFC. This technology is safe because the small distance range in which it operates makes it very difficult to sniff part of the communication by third parties. We assume then that special security protocols are not required; however, an algorithm that verifies consistency and integrity of vouchers has been used to improve the strength of the system.

The method is similar to the one used in certificates and digital signatures. When a user downloads a voucher, the server sends all the information of the voucher and also a digest codified as a byte array. The contents of the digest are hidden by the encryption with a symmetric key algorithm. The algorithm used is Blowfish [54], and it uses a private key that is hosted on the server. Users do not need to know the information encoded in the digest, because it is not needed to expose the key. Thus, each time the user downloads a voucher the application stores data corresponding to the voucher and an array of bytes, which encodes the encrypted digest.

When the user wants to perform voucher redemption with NFC, the mobile application transmits all the voucher data to the controller installed on the store. This requires sending the server all the received data with the purpose of verifying the authenticity of the voucher. Once the data, originally sent by the store, is in the server, it decrypts the digest and verifies that the digest corresponds exactly to the provisioned voucher data. The server also checks the identification of the user involved in the process testing if he/she is the voucher owner. If integrity is maintained and the voucher is valid, the server sends a confirmation message to the controller; otherwise it sends an error message.

When the redemption is made using QR codes, the procedure is similar because the code has enough storage capacity to store the voucher data and the encrypted digest. The redemption of vouchers with bar codes does not support this verifying method, and therefore its use is not recommended.

Finally, transference of vouchers between users can only be performed with secure vouchers and indentified users. This process is always validated by WingBonus server checking the user and vouchers authenticity and the rules defined for those vouchers in the marketing campaign.

### 3.5. Synchronization Process

Synchronization is a process performed by the mobile application automatically or on user's demand. This process is in charge of several important actions (see Figure 3):

– Updating the mobile database with information corresponding to the selected voucher from the WingBonus and provider's websites, sourcing the vouchers and updating the server database.
– Sending the server partial information of vouchers gathered in the selection process from smart posters or shops. Vouchers are updated with information, stored in the server database and sent back to the mobile device in order to update the mobile database.
– Matching information corresponding to mobile and server databases is analyzed in order to detect errors, lacks, inconsistencies or counterfeits. In this process, entries in the mobile database are sent to the server to be validated and send back to the mobile. Vouchers accidentally lost by the user are then recovered.

Sending and receiving information between the mobile and server sides has been implemented by encapsulating information in JSON objects. Unlike the usual format, web services have been used in both directions: to initiate and to finish the communication.

Synchronization process requires identification of the user. Although anonymous users can select, source and even redeem unsecure vouchers, during the synchronization, the user must be authenticated through a login process.

## 4. WingBonus Website

The WingBonus server subsystem is composed of several functional components: (a) the website responsible for the web interaction with the user and the administrators, (b) the database storing all the information involving users, vouchers, clients, establishments, *etc.*, and (c) a set of web services that compose an API that is consumed by the mobile application and the web-client side.

The WingBonus server has been built using the one-page paradigm, using Backbone.js that supports Model-View-Controller (MVC) [62], HTML5 and CSS3 for the page makeup and JQuery for DOM manipulation. All the work concerning interactions with the final user is made on the client side once the website has been loaded, letting the server be an API and a set of services that work against the database. Like other JavaScript web applications, WingBonus uses several libraries such as Underscore, Require.js, *etc.*

**Figure 4.**
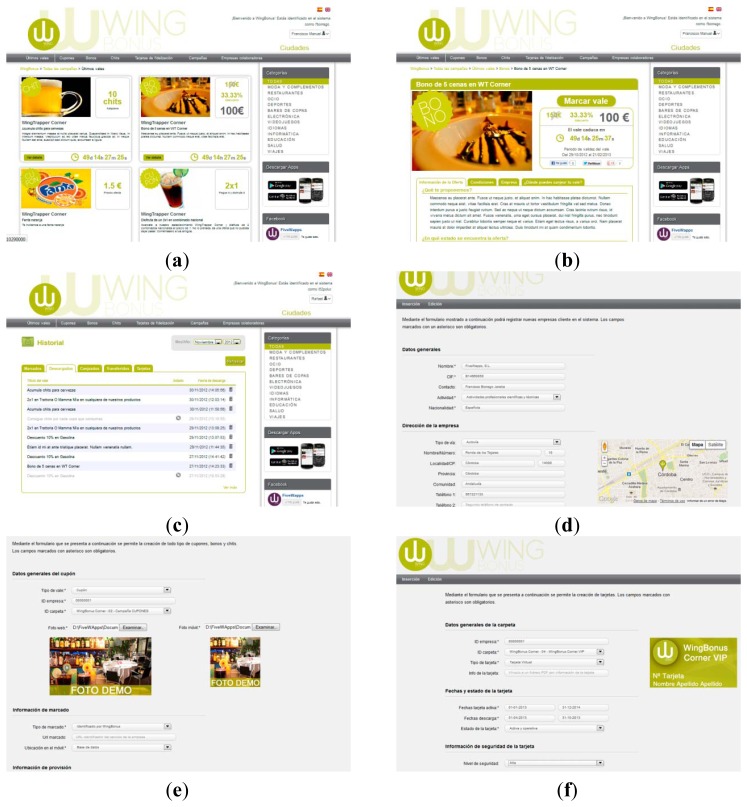
Some snapshots of the WingBonus website.

The WingBonus website constitutes an access platform for users and system administrators. From the main page, shown in Figure 4(a), users can access the registration form. Once registered, the user can define a set of profile options that allows WingBonus to build a personal site showing information tailored to the user.

Users can view offers classified in different items: coupons, bonds and chits. In addition, offers can be sorted by companies and marketing, simply by selecting the desired option in the upper selection bar. In addition, the voucher view can be filtered by categories selected from the right side selector.

Once a voucher has been selected, a detailed view is shown as in Figure 4(b). Then, the user can “mark” the voucher to be later provided in the next synchronization process from the mobile application.

The full user activity is stored in the database. Thus, security of marked and provisioned vouchers can be checked before the exchange process. In addition, the server system provides information about the user activity. As Figure 4(c) shows, users can view the whole history of their related vouchers.

Moreover, the WingBonus server provides a complete administration tool for the management of the information. Thus, Figure 4(d) shows the screenshot corresponding to the management of user data for retailers/providers. Figure 4(e) corresponds to the management of an advertising campaign for vouchers and Figure 4(f) to the management of loyalty cards. In addition, this administration tool involves a set of functionalities in charge of the data analysis and of generating different reports oriented to collecting information on users, vouchers, user preferences and activity, marketing, companies and so on.

As mentioned previously, neither the mobile application nor the JavaScript part of the website should access the database directly. For this purpose, we have developed an API for performing any operation needed for the database; giving in addition facilities for logging, registration and user activation, as well as voucher retrieving. Thus, the information is protected as a layer over the database, normalizing the access and allowing the adaptation of the input/output to each scenario. The API acts on the server side and has been developed using PHP and giving outputs formatted as a JSON object.

The web services provided by the API also agglomerate the operations for the synchronization. Generally, this kind of system gives an output based on a few inputs. WingBonus implements a complete system that receives all the data to be updated from the mobile application and operates over the database. Later, the services give a JSON response with the data requested by the mobile application, letting the device updated and fixing the date of the synchronization process.

## 5. WingBonus Mobile Application

The WingBonus mobile application allows checking and downloading vouchers anywhere at any time, with the purpose of carrying them in the phone and solving the problem of paper coupons or loyalty cards (forgetting them or loss). Besides this, the mobile application allows getting vouchers by means of smart posters and using it with the advantages that NFC technology provides, or QR codes in case NFC is not supported by the phone device. The mobile application has been developed for the Android 2.3 (or later) and RIM 7.1 operating systems.

**Figure 5.**
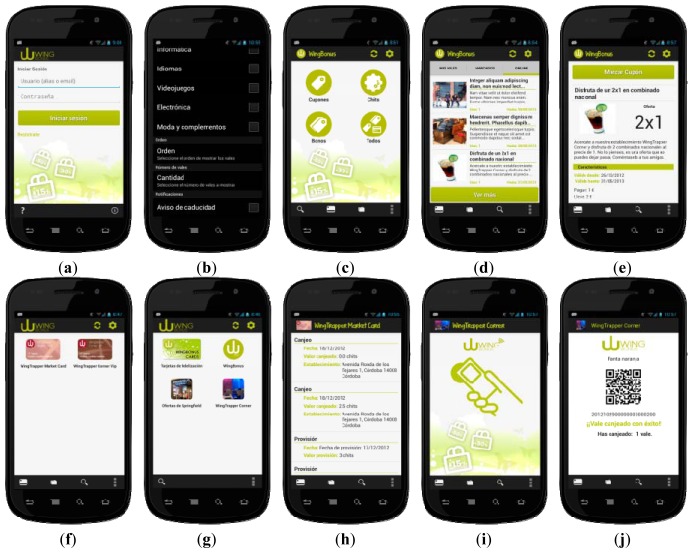
Some WingBonus mobile application snapshots.

The first time the user runs the application, the screen of Figure 5(a) is shown. The application distinguishes between anonymous and registered users. If the user is registered, he/she has to introduce his/her credentials on the login form. Anonymous users have limited use while authenticated users can access the whole system functionality without restrictions. Both types of users can customize the application through the preference windows (see Figure 5(b)), different options such as, the number of coupons shown, the sorting order, favorite categories and activate nearness and expiration notifications of vouchers, can be set up.

Once the user is identified (being registered or anonymous), the mobile application shows the main menu (Figure 5(c)). From this menu, users can perform any operation implemented in the WingBonus system. Thereby, users can download coupons (see Figure 5(d)), view a single coupon detail (Figure 5(e)), remove coupons, use a coupon or download a loyalty card (Figure 5(f)) among other actions.

WingBonus mobile allows storing an unlimited number of electronic vouchers. As Figure 5(g) shows, vouchers are organized in containers, identifying the different provider campaigns or companies. In addition, containers could be organized in folders. Folders indentify the different business activities and different campaigns using different images and they may be assigned different requirements or characteristics depending on company politics.

By default, the application installs the WingBonus container. This container can never be deleted. Installation of any other container or folder is automatically performed during the synchronization process when a voucher belonging to the container or folder is provisioned. Loyalty cards also are stored and shown to the users in the folders (see Figure 5(f)). Both containers and folders are easily accessible from the main menu.

Mobile application permits the user to view the existing voucher offers that fits the user preferences (see Figure 5(b)). Selected and marked vouchers are temporally stored in the mobile database, until in the next secure synchronization process the vouchers are checked against the server database and provisioned to the mobile database.

Although in some synchronizations the amount of transferred information between mobile and server applications may be small (few number of vouchers), usually the synchronization process will require the exchanged of new preferences, download and upload of vouchers, *etc.* In any case, the time required is negligible. During this process, all user data is checked on the server, and fake or inconsistent vouchers are erased.

In addition, vouchers can also be acquired through smart posters. The storage capacity of an RFID tag is enough to store a complete electronic voucher. Two types of supplying are possible using smart posters:

– The smart poster has a tag storing a “mark record”. When the user touches the tag, the record is transferred via NFC and stored in the mobile database. Then, the record is used to download the voucher as if it had been marked directly from the Website,
– The tag stores the provisioned voucher, so the user will receive the full voucher information without a further synchronization against the server. This last type of voucher is only implemented for unsecured vouchers or in case the provider wants the broadcasting of an offer without control.

How offers are shown is completely dependent on user preferences. User can view a summary of stored vouchers with the possibility to access the detailed information, as shown in Figure 5(h).

The most important feature of WingBonus is the secure exchange of coupons using NFC (Figure 5(i)). A voucher that has been already provided and stored in the mobile phone database can be exchanged selecting the appropriate option from the detailed view of such voucher. The mobile application supports two ways to redeem vouchers:

– The SNEP Specification (NFC Simple NDEF Exchange Protocol) [63] allows an application on an NFC-enabled device to exchange NFC Data Exchange Format (NDEF) messages with another NFC Forum device when operating in NFC Forum peer-to-peer mode. The protocol makes use of the Logical Link Control Protocol (LLCP) connection-oriented transport mode to provide a reliable data exchange. Using the SNEP in order to send the information of the voucher to the NFC reader, and wait for a reply that is sent from the server to the mobile phone through the NFC reader in order to complete the P2P communication. By using this technology, users can also exchange vouchers between them. Thus, the user sends the voucher identification to another user by NFC P2P. Using this identification, the second user will download the transferred voucher. A confirmation signal is sent back by the server to both users for accepting or cancelling the process.
– Although the ideal exchange method is NFC, redemption can be performed through QR codes and traditional barcodes. WingBonus uses the ZXing [64] core, in order to generate the QR barcode that contains all the information needed to redeem the voucher (Figure 5(j)). This QR code is shown by the mobile application and read by the barcode reader. The information read is taken by the controller and sent securely to the server, which is responsible for checking the integrity. If the information is properly verified, a confirmation message is displayed on the controller and the voucher is redeemed.

WingBonus mobile also supports traditional barcode formats like EAN13. These codes can save thirteen characters, some of which are reserved for convention, allowing a minimum storage capacity. The store can read the barcode and redeem the voucher, but the server cannot verify the integrity. This procedure is maintained to facilitate the integration of the application on the market but is strongly discouraged.

## 6. Discussion and Remarks

The combination of NFC with the growing success of mobile technology will allow, in the near future, the development of “ideally” smart environments, which were conceived decades ago. The application of NFC to marketing models of companies offers several advantages over existing systems. There are several and important companies that provide discount and offer services, however, getting and using coupons is not always easy. The main weak point is the client's interaction with the coupon and the company or system that serves the product or service. The use of NFC and its application in mobile devices provides users with an easy way to acquire, store, manage and use vouchers. Besides, these features make the process fast, secure, efficient and transparent.

The system described in this paper has the advantage of the consideration of any type of vouchers: discount coupons, bonds and chits. In addition, loyalty cards are also considered and the card operations such as chit operations are managed. Managing marketing campaigns in containers and folders, WingBonus provides a powerful diffusion tool for companies and their marketing activities, as well as an easy way for the users to manage offers.

In addition, WingBonus can be fully tailored to different marketing strategies. Thus, each campaign and type of voucher can be adapted to a different security level, source of distribution and retailer infrastructure. This versatility allows its application for companies of different size and commercial sector.

WingBonus allows consumers to take advantage of all offers made by companies. This guarantees a huge savings on the purchase of consumer products. Vouchers and loyalty cards are carried on the mobile phone without any possibility of fraud, loss or forgetting.

For partner companies, WingBonus offers great advantages: cost reductions, elimination of paper and/or plastic supports, reaching more customers, elimination of forgeries, real time tracking, market analysis, study of trends, capability to build customer loyalty, *etc.* Moreover, the commerce where the vouchers are adopted benefits from the speed and safety of the process.

Currently, we are working on the improvement of the system described in this paper in order to add new features and also allow the storage of the offers in the secure element of the NFC phone. In addition, we are combining the marketing based on vouchers and loyalty cards with marketing based on gaming, thus offering new models for attracting loyal customers to companies.
